# Association of PECAM-1 Gene Polymorphisms with Kawasaki Disease in Chinese Children

**DOI:** 10.1155/2017/2960502

**Published:** 2017-04-23

**Authors:** Zhuoying Li, Dong Han, Jie Jiang, Jia Chen, Lang Tian, Zuocheng Yang

**Affiliations:** Department of Pediatrics, The Third Xiangya Hospital of Central South University, Changsha, Hunan 410013, China

## Abstract

Kawasaki disease (KD) is an acute systemic vasculitis complicated by development of coronary artery lesions. PECAM-1 is a kind of cell adhesion molecule, which plays an important role in coronary artery disease. The relationship between PECAM-1 gene polymorphisms and their susceptibility to Kawasaki diseases (KD) is still unclear. In our study, we examined the PECAM-1 gene polymorphisms in 44 KD patients and 59 healthy children and revealed the correlation of PECAM-1 gene polymorphisms in KD children with and without coronary artery lesions (CAL).

## 1. Introduction

Kawasaki disease (KD), also known as acute febrile mucocutaneous lymph node syndrome, is characterized by systemic febrile vasculitis symptoms of an unknown etiology occurring mostly in infants or young children [1, 2]. KD may be attributed to an innate immune disorder and the injury of vascular endothelial cells caused by large numbers of inflammatory mediators and cytokines [3–6]. The etiology of KD is still unclear and no consistent etiological agent for KD has been identified yet. Although to date, its etiology remains unknown, various findings suggested that genetic polymorphisms show a close relationship with the susceptibility of KD. Platelet endothelial cell adhesion molecule-1 (PECAM-1) is an important negative adhesion regulator for accumulation platelets and plays an important role in signal transduction and inflammation. The correlation between PECAM-1 polymorphism with multiple clinical diseases such as septic shock, cerebral infarction atherosclerotic, and CHD has been proved [7–9]. Many researches revealed that various genes polymorphisms are involved in susceptibility to KD such as IL-10 [10, 11], IL-31 [12], SRC-1 [13], TIAM1 [14], SMAD5 [15], CD40 [16], and CTLA-4 [17]. But no study has identified the possible association between PECAM-1 gene polymorphism and the development of Kawasaki diseases with or without CAL.

## 2. Materials and Methods

### 2.1. Subjects

A total of 113 individuals (44 patients with KD and 59 healthy controls) were enrolled from January 2006 to August 2010 in Xiangya Third Hospital, Changsha, China. And the number of KD patients is accounting for 0.43% of the total 10092 patients. All KD patients met the diagnostic criteria of the Japanese Kawasaki Disease Research Committee [18]. The patients all met at least 5 of the following clinical symptoms: (1) an unknown fever cause lasting 5 d or more, (2) bilateral conjunctival injection, (3) changes in lips or oral cavity (e.g., pharyngeal erythema, dry/fissured or swollen lips, and strawberry tongue), (4) changes in extremities (e.g., erythema, edema, and desquamation), (5) polymorphous rash, and (6) cervical lymphadenopathy, diameter ≥ 1.5 cm. Moreover, with coronary artery aneurysm or coronary expansion detected by echocardiography or coronary angiography, the patients that were recruited for this study met above 4 symptoms. All patients were Han Chinese individuals aged from 7 months to 7 years. There were 30 males and 14 females, with an average age at onset of 2.4 ± 1.4 years, and 63.6% of them had CAL, which was defined as having a diameter of >2.5 mm of either the left or right coronary artery in patients < 3  years of age, a diameter of or >3 mm in patients < 5  years of age, a diameter of or >3.5 mm in patients < 14  years of age, or a diameter that was >1.5 times than that of an adjacent vessel, and the vessel wall was irregular. A total of 59 healthy controls (30 males and 29 females; average age: 3.8 ± 1.9 years) were recruited from the Xiangya Third Hospital during the same period. They were children aged from 9 months to 9 years, who never had any previous history of KD, infectious diseases, cardiovascular disease, and rheumatic diseases. The sex composition and age distribution of the two groups were not statistically significant (*P* > 0.05). The clinical characteristics of the KD patients and the control subjects are shown in Table 1. To further investigate the relationship between Kawasaki disease and CAL, the Kawasaki disease group was divided into the CAL group and the non-CAL group. Informed consent was obtained from the parent or legal guardian of each patient according to the Declaration of Helsinki guidelines. The study and study protocol were approved by the Ethics Review Committee of Medical Research Institute and Institutional Review Boards of the Medical Research Institute at the Xiangya Third Hospital.

We collected blood samples from KD patients and cohort controls in disposable blood collection tubes (containing anticoagulant EDTA-K2). Blood samples were immediately transferred to cryopreservation tubes and stored at −80°C. Genomic DNA was extracted and purified from the whole blood samples of all subjects by use of PUREGENE DNA purification kits according to the manufacturer's instructions (Gentra, USA).

### 2.2. Polymorphism Genotyping

The detection of genotypes of PECAM-1 SNP 373 C/G (dbSNP ID: 668) and 1688 A/G (dbSNP ID: 17354990) was performed by polymerase chain reaction-restriction fragment length polymorphism (PCR-RFLP). We amplified the polymerase chain reactions (PCR) of the PECAM-1 373 C/G polymorphism, using forward primer 5-GCA CCA CCT CTC ACG TCA AG-3 and reverse primer 5-CTG TGC TCA GTT CCA AGG AC-3′. And PECAM-11688 A/G was amplified using forward primer (5′-TGT TCC TTT TCC TGC TTT TC-3) and reverse primer (R: 5-GCT TGC TAT GGA GAC CCT GAC-3). All the primers were synthesized by the Shanghai Biological Engineering Company. The PCR reactions were carried out in reaction mixes containing 2 *μ*l diluted gene template, 10 *μ*l Premix Taq Master Mix, 2 *μ*l primer, and ddH_2_O to a final volume of 20 *μ*l. The PCR program was as follows: initial denaturation at 95°C for 5 min, followed by 35 cycles at denaturation at 94°C for 1 min, annealing at 62°C for 1 min, extension at 72°C for 5 min, and then performed a final extension step at 72°C for 15 min. The PCR products were then digested with restriction enzyme Pvu II at 37°C for 16 h after its quality was examined by 2% agarose gel electrophoresis. The digested reaction was performed in a 20 *μ*l reaction mixture containing 7 *μ*l ddH_2_O, 2 *μ*l Buffer G, 10 *μ*l DNA amplification products, and 1 *μ*l endonuclease (enzymes for 373 C/G site is Pvu II and 1688 A/G is Nhe I, Fermentas, USA). The digested products were electrophoresed in a 2.0% agarosegel along with a molecular marker.

### 2.3. Statistical Analysis

Statistical analyses were performed with SPSS version 17.0 (SPSS Inc., Chicago, IL, USA). All of the allele frequencies were in line with the Hardy-Weinberg equilibrium. The direct counting method was used to calculate the allele and genotype frequencies. The chi-square test was used to examine the differences in genotype and allele frequencies between the two groups. The odds ratio (OR) along with its 95% confidence interval (95% CI) were estimated for associations between risk alleles and genotypes with KD. All tests were two sided and the probability of less than 0.05 was considered as statistically significant.

## 3. Result

### 3.1. Amplification Results of Gene Polymorphism

**Figure 1:**
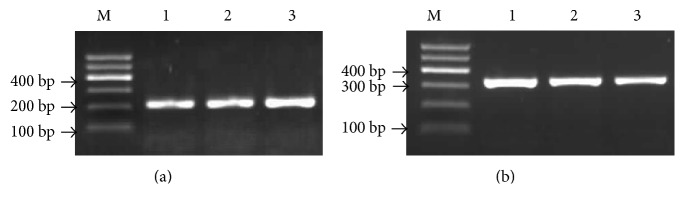
Representative results of the two PECAM-1 polymorphism sites in two groups. Lane M, DNA size marker. Lane 1, KD without CAL; Lane 2, KD with CAL; Lane 3, control's sample. (a) PECAM-1 +373 C/G gene. (b) PECAM-1 +1688 A/G gene.

DNA extracted from the whole blood is detected by NanoDrop 2000 UV-Vis spectrophotometer. The result showed that the OD 260/280 ratio of the genomic DNA were all approximately between 1.7 and 2.0 that meets the requirement of PCR amplification. The +373 C/G gene fragment of PECAM-1 gene was 225 bp amplified by polymerase chain reaction-restriction fragment polymorphism (PCR-RFLP), and the 1688 A/G gene fragment was 338 bp. The molecular assay of the two selected SNPs of PECAM-1 was displayed in the gel (Figure 1).

We analyzed the association between the PECAM-1 gene SNPs and their susceptibility to KD by using the logistic regression model. No significant deviations from the Hardy-Weinberg equilibrium were observed in PECAM-1 +373 C/G (KD: *χ*^2^ = 1.33, *P* > 0.05; control: *χ*^2^ = 0.77, *P* > 0.05) and PECAM-1 gene +1688 A/G (KD: *χ*^2^ = 2.32, *P* > 0.05; *χ*^2^ = 1.44, *P* > 0.05). These results suggested that the subjects involved in this study were from large groups, and the individuals were randomly distributed, which indicated that our research was performed with high sensitivity of the whole cohort.

### 3.2. Association of PECAM-1 (+373 C/G) Polymorphisms with KD

**Figure 2:**
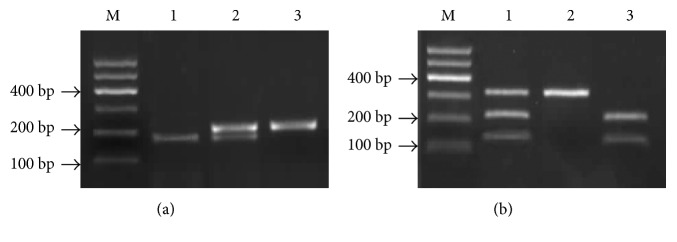
PCR-RFLP assay for PECAM-1 gene (+1688 A/G) and (+373 C/G) polymorphism. Representative electrophoresis of PCR products cleaved with restriction endonuclease Pvu II and Nhe I was shown. Lane M: DNA molecular weight marker. (a) PECAM-1 (+373 C/G) gene. Lane 1: 373CC homozygous genotype, 188 bp; Lane 2: 373CG heterozygous genotype, 255 bp and 188 bp; Lane 3: 373GG homozygous genotype, 255 bp. The 37 bp in 373 CC and 373 CG genotypes was not visible in electrophoresis gel due to its too small size. (b) PECAM-1 (+1688 A/G) gene. Lane 1: 1688AG heterozygous genotype, 338 bp, 219 bp, and 119 bp; Lane 2: 168AA homozygous genotype, 338 bp; and Lane 3: 1688GG homozygous genotype, 219 bp and 119 bp. PECAM-1: platelet endothelial cell adhesion molecule-1.

The genetic association between the two SNPs of PECAM-1 gene and their susceptibility to KD was investigated. The study enrolled 44 KD patients and 59 healthy control subjects. The clinical features of the KD patients and the control subjects are shown in Table 1. The 44 KD patients included 26 patients with CAL and 18 patients without CAL. At +373 C/G of PECAM-1, there is a Pvu II restriction site in 373 C allele but not in 373 G allele. PCR product digestions with Pvu II were performed to enable restriction site distribution analysis. Electrophoretic analysis has shown that there is 1 band for GG genotype (255 bp), 2 bands for CC genotype (188, 37 bp), and 3 bands for CG genotype (255 bp, 188 bp, and 37 bp) (Figure 2(a)). The 37 bp fragment was too small to be visible in the electrophoresis gel. In genotypic analysis, there was no significant difference in C and G allele frequency of PECAM-1 373 C/G in the KD group compared with that in the normal control group (*χ*^2^ = 1.11, *P* > 0.05). While the genotype distribution of CC, GG, and CG was significantly different in the Kawasaki disease group (*χ*^2^ = 8.49, *P* < 0.05), CG genotype distribution is significantly higher in the KD group than in the control group (OR = 2.739; 95% CI: 1.222–6.139; *P* = 0.013) (Table 2). This finding suggests that the CG genotype of PECAM-1 +373 C/G may be related to increased susceptibility to KD in Chinese children.

### 3.3. PECAM-1 (+1688 A/G) Polymorphism in KD and Control Subjects

The G allele in PECAM-1 (+1688 A/G) contained one Nhe I restriction site, while the A allele did not contain the Nhe I restriction site; so, the electrophoresis banding pattern of PCR products is that AA genotype shows one band (338 bp), GG genotype has two bands (219 and 119 bp), and AG genotype possesses three bands (338, 219, and 119 bp) after Nhe I digestion (Figure 2(b)). The genomic frequencies of the PECAM-1 +1688 A/G polymorphism in children with KD were 25% (11/44) for AA, 61.4% (27/44) for CG, and 13.6% (6/44) for GG. In controls, these frequencies were 25.4% (15/59) for AA, 57.6% (34/59) for CG, and 17% (10/59) for GG. The PECAM-1 +1688 A/G polymorphisms tested in this study failed to show any significant associations with genotype or allele frequency in KD patients compared with those of controls (genotype: *χ*^2^ = 0.24, *P* = 0.887; allele frequency: *χ*^2^ = 0.04, *P* = 0.836) (Table 3).

### 3.4. Association of PECAM-1 +373 C/G and +1688 A/G Polymorphism with CAL Formation in KD Patients

Further analysis found that KD patients with the PECAM-1 (+373 C/G) GG and CC genotypes had a higher rate of CAL formation (*P* = 0.045) than those with the CG genotype (Table 4). While there is no significant difference for the PECAM-1 (+373 C/G) in allele frequency between CAL in the Kawasaki disease group (+373 C/G: *χ*^2^ = 5.19, *P* = 0.0745). PECAM-1 (+1688 A/G) had no significant association with CAL formation among KD patients (genotype distribution: *χ*^2^ = 0.376, *P* = 0.828; allele frequency: *χ*^2^ = 0.0004, *P* = 0.984). These results suggest that homozygous GG genotype of PECAM-1 (+373 C/G) is associated with CAL formation among KD patients; the PECAM-1 (+1688 A/G) polymorphism shows no significant association with the development of CAL in Chinese children with KD.

## 4. Discussion

Kawasaki disease (KD) is an acute vasculitis with unknown fever causes, which affects the children under 5 years old [12]. KD has a predilection for the small-to medium-sized arteries, especially the coronary arteries. For some serious KD patients, it even developed coronary artery lesions (CAL), including aneurysm formation [2, 19]. Despite the several decades of extensive study for the etiology of KD, its pathogenesis is still unclear [2, 20]. It has been reported that these pathways included complement activation, coagulation, and *Staphylococcus aureus* infection, and response to elevated platelet cytosolic Ca^2+^ was involved in the pathogenesis of KD. Recently, more and more genetic investigations have revealed that single-nucleotide polymorphisms (SNP) are associated with KD. PECAM-1 (also called CD31) is a major transmembrane glycoprotein, which belongs to the immunoglobulin superfamily, and mainly expressed on the surface of the blood and vascular cells [21, 22]. The PECAM-1 is an adhesion molecule that plays a vital role in vascular biology. Previous studies reported that PECAM-1 polymorphisms have been associated with coronary artery stenosis or atherosclerosis [23–25]. Three common polymorphisms (Leu125Val, Asn563Ser, and Gly670Arg) located on the PECAM-1 gene have been verified to be in association with these diseases [8, 24, 26]. And it found that +373 C/G (Leul25Val) polymorphism is associated with cerebral infarction or myocardial infarction [26, 27]. PECAM-1 gene +373 C/G (Leul25Val) mutation position is in Exon-3 encoding first-extracellular Ig-like domain [26, 28]. PECAM-1/PECAM-1 homophilic binding capability and leukocyte emigration, the two main biology of PECAM-1, are in association with gene locus. When +373 C/G (Leul25Val) mutated that will cause the amino acids of PECAM-1 protein to change, then PECAM-1 adhesion activity will be changed. The adhesion effect of PECAM-1 mediates leukocyte migration by activating integrin *β*1, *β*2h, and *β*3. Integrins or proteases released by the stimulation of PECAM-1 and its heterophilic activity, which allowed leukocytes to transmigrate through basement membrane, will change the properties of endothelial cells. That is a foundation for the subsequent vascular endothelial injury. It is reported that subjects with the homozygous GG genotype of the Leu125Val polymorphism had higher serum soluble PECAM-1 (sPECAM-1) levels [29]. In addition, the polymorphism of Leul25Val of PECAM-1 gene was associated with coronary artery ectasia [29, 30]. Our results suggested that the frequencies of CC, GG, and CG genotypes of PECAM-1 gene 373 C/G in the Kawasaki disease group were statistically different from those in the normal control group. The heterozygote CG genotype distribution of PECAM-1 +373 C/G was significantly higher compared with homozygotes CC and GG in the two groups. High frequency of CG genotype in PECAM-1 +373 C/G may be associated with susceptibility to KD in Chinese children. However, the difference of allele frequencies at PECAM-1 +373C/G was not statistically significant in the two groups. The 373GG genotype of PECAM-1 (+373 C/G) at this polymorphism also presents a significant difference between KD children with and without CAL. So, the CG genotype polymorphism of PECAM-1 +373 C/G is closely related with KD and 373GG homozygous genotype is associated with the formation of CAL in the KD group.

1688 A/G (Ser563Asn) is expressed in exon 8 and located on the sixth Ig-like homology domain of PECAM-1 that plays an important role in the heterophilic interaction with integrins [28, 31]. Substituting an amino acid may influence the binding affinity with integrins and the function of cell signal transduction, which caused an increase of soluble PECAM-1 by a positive feedback regulation of the expression of PECAM-1 in the nucleus [21, 31, 32]. Then, PECAM-1 mediated leukocytes binding with vascular endothelial cell in the blood. The adherent leukocytes migrate across the endothelium, accumulate in the subendothelial space, and turn into foam cells. The above processes increased the inflammatory response of the blood vessels and resulted in coronary artery disease and stroke events. Systemic vasculitis is the main pathological properties of Kawasaki disease, also associated with CAL. Owing to the important role of 1688 A/G (Ser563Asn) in the vascular biology function, we hypothesized that this polymorphism might be associated with Kawasaki disease. Unexpectedly, the genomic and allelic frequencies at this polymorphism did not significantly differ between KD children and control subjects. Unfortunately, there was no statistically significant difference in the frequencies of genotype and allele of PECAM-1 gene 1688 A/G between patients with CAL and those without CAL. These results suggested that PECAM-1 gene 1688 A/G polymorphism may not be associated with Kawasaki disease and the development of CAL in KD. The genomic and allelic frequencies at this polymorphism did not significantly differ between children with KD and control subjects. In addition, there was no association between the PECAM-1 +1688 A/G gene polymorphism and development of CAL in the enrolled KD patients.

In conclusion, this study demonstrates for the first time that PECAM-1 +373 A/G gene polymorphism is associated with the presence of KD and may be a genetic risk factor for the development of CAL in Chinese children. Although Asn563 in the Ig6 domain of PECAM-1 may be a genetic risk factor for development of coronary heart disease, there is no association with the susceptibility of KD and the development of CAL in this gene site. This work will provide important new insights into the KD pathology. However, for the limited samples, future analysis of a larger KD patient group will be needed to verify the relationships between PECAM-1 gene polymorphisms and their susceptibility to KD.

## Figures and Tables

**Table 1:** Demographics of patients with Kawasaki disease and control subject.

	KD			Control subjects
	With CAL	Without CAL	Total	
Number of patients	26	18	44	59
Gender (M/F)	17/9	9/9	30/14	30/29
Age (years)	2.61 ± 1.71	2.15 ± 1.52	2.4 ± 1.4	3.8 ± 1.9

**Table 2:** PECAM-1(+373 C/G) genotype and allele frequency in KD patients and controls.

		KD (*n* = 44) *n* (%)	Control (*n* = 59) *n* (%)	*P* value	OR (95% CI)
Genotypes	CC	4 (9.1)	17 (28.8)	**0.017**	
	GG	12 (27.3)	19 (32.3)		
	CG	28 (63.6)	23 (38.9)		

Genotype comparison	CC+GG	16 (36.4)	36 (61.1)	**0.013**	2.739 (1.222–6.139)
	CG	28 (63.6)	23 (38.9)		

Allele	C	36 (40.9)	57 (48.3)	0.2913	0.741 (0.424–1.294)
	G	52 (59.1)	61 (51.7)		

**Table 3:** PECAM-1 (+1688 A/G) genotype and allele frequency in KD patients and controls.

		KD (*n* = 44) *n* (%)	Control (*n* = 59) *n* (%)	*P* value	OR (95% CI)
Genotypes	AA	11 (25.0)	15 (25.4)	0.887	
	GG	6 (13.6)	10 (17.0)		
	AG	27 (61.4)	34 (57.6)		

Genotype comparison	AG	27 (61.4)	34 (57.6)	0.703	1.168 (0.526–2.591)
	AA+GG	17 (38.6)	15 (42.4)		

Allele	C	49 (55.7)	64 (54.2)	0.836	1.060 (0.609–1.847)
	G	39 (44.3)	54 (45.8)		

**Table 4:** Genotype and allele frequencies of polymorphisms of the PECAM-1 gene in the KD group with CAL and without CAL.

cSNP	Genotype allele	KD with CAL (*n* = 28) *n* (%)	KD without CAL (*n* = 16) *n* (%)	*P* value	OR (95% CI)
+373 C/G	CC	3 (11.5)	1 (5.6)	0.074	
	GG	10 (38.5)	2 (11.1)		
	CG	13 (50.0)	15 (83.3)		
	GG	10 (50)	2 (16.7)	**0.045**	5.000 (0.942–26.54)
	CC+CG	16 (50)	16 (83.3)		
	C	19 (36.5)	17 (47.3)	0.316	0.643 (0.271–1.527)
	G	33 (63.5)	19 (52.7)		

+1688 A/G	AA	7 (26.9)	4 (22.2)	0.828	
	GG	4 (15.4)	2 (11.1)		
	AG	15 (57.7)	12 (66.7)		
	AA+GG	11 (42.3)	6 (33.3)	0.548	0.682 (0.195–2.384)
	AG	15 (57.7)	12 (66.7)		
	C	29 (55.8)	20 (55.6)	0.984	1.009 (0.428–2.373)
	G	23 (44.2)	16 (44.4)		

## References

[1] Peng Q., Deng Y., Yang X., Leng X., Yang Y., Liu H. (2016). Genetic variants of ADAM17 are implicated in the pathological process of Kawasaki disease and secondary coronary artery lesions via the TGF-β/SMAD3 signaling pathway. *European Journal of Pediatrics*.

[2] Kuo H. C., Chang J. C., Guo M. M. (2015). Gene-gene associations with the susceptibility of Kawasaki disease and coronary artery lesions. *PloS One*.

[3] Hara T., Nakashima Y., Sakai Y. (2016). Kawasaki disease: a matter of innate immunity. *Clinical and Experimental Immunology*.

[4] Ma L., Du Z. (2016). Advances in the pathogenesis of vascular endothelial cells injury in Kawasaki disease. *Zhonghua Er Ke Za Zhi*.

[5] Tian J., Lv H. T., An X. J., Ling N., Xu F. (2016). Endothelial microparticles induce vascular endothelial cell injury in children with Kawasaki disease. *European Review for Medical and Pharmacological Sciences*.

[6] Lin Y. J., Chang J. S., Liu X. (2014). Genetic variants of glutamate receptor gene family in Taiwanese Kawasaki disease children with coronary artery aneurysms. *Cell & Bioscience*.

[7] Sun W., Li F. S., Zhang Y. H., Wang X. P., Wang C. R. (2015). Association of susceptibility to septic shock with platelet endothelial cell adhesion molecule-1 gene Leu125Val polymorphism and serum sPECAM-1 levels in sepsis patients. *International Journal of Clinical and Experimental Medicine*.

[8] Song Y., Li Q., Long L., Zhang N., Liu Y. (2015). Asn563ser polymorphism of CD31/PECAM-1 is associated with atherosclerotic cerebral infarction in a southern Han population. *Neuropsychiatric Disease and Treatment*.

[9] Xia T., Liu X., Du C. J., Jin X., Kong X. Q., Li G. (2015). Association of Leu125Val polymorphisms in the PECAM-1 gene with the risk of coronary heartdisease: a meta-analysis. *International Journal of Clinical and Experimental Medicine*.

[10] Weng K. P., Hsieh K. S., Hwang Y. T. (2010). IL-10 polymorphisms are associated with coronary artery lesions in acute stage of Kawasaki disease. *Circulation Journal*.

[11] Hsieh K. S., Lai T. J., Hwang Y. T. (2011). IL-10 promoter genetic polymorphisms and risk of Kawasaki disease in Taiwan. *Disease Markers*.

[12] Tseng W. N., Lo M. H., Guo M. M., Hsieh K. S., Chang W. C., Kuo H. C. (2014). IL-31 associated with coronary artery lesion formation in Kawasaki disease. *PloS One*.

[13] Chen Y. T., Liao W. L., Lin Y. J., Chen S. Y., Tsai F. J. (2014). Association between SRC-1 gene polymorphisms and coronary artery aneurysms formation in Taiwanese children with Kawasaki disease. *Journal of Clinical Laboratory Analysis*.

[14] Wang X., Zhu T. J., Zhou X. F., Wan Z. T. (2015). Association of TIAM1 gene polymorphisms with Kawasaki disease and its clinical characteristics. *Zhongguo Dang Dai Er Ke Za Zhi*.

[15] Cho J. H., Han M. Y., Cha S. H., Jung J. H., Yoon K. L. (2014). Genetic polymorphism of SMAD5 is associated with Kawasaki disease. *Pediatric Cardiology*.

[16] Cheng S. C., Cheng Y. Y., Wu J. L. (2014). Association between gene polymorphism of CD40 gene and coronary artery lesion in Kawasaki disease. *Zhongguo Dang Dai Er Ke Za Zhi*.

[17] Kuo H. C., Liang C. D., Yu H. R. (2011). CTLA-4, position 49 A/G polymorphism associated with coronary artery lesions in Kawasaki disease. *Journal of Clinical Immunology*.

[18] Yanagawa H., Nakamura Y., Yashiro M., Uehara R., Oki I., Kayaba K. (2006). Incidence of Kawasaki disease in Japan: the nationwide surveys of 1999-2002. *Pediatrics International*.

[19] Zhang X., Liang Y., Feng W., Su X., Zhu H. (2016). Epidemiologic survey of Kawasaki disease in Inner Mongolia, China, between 2001 and 2013. *Experimental and Therapeutic Medicine*.

[20] Uehara R., Belay E. D. (2012). Epidemiology of Kawasaki disease in Asia, Europe, and the United States. *Journal of Epidemiology*.

[21] Privratsky J. R., Newman P. J. (2014). PECAM-1: regulator of endothelial junctional integrity. *Cell and Tissue Research*.

[22] Torzicky M., Viznerova P., Richter S. (2012). Platelet endothelial cell adhesion molecule-1 (PECAM-1/CD31) and CD99 are critical in lymphatic transmigration of human dendritic cells. *The Journal of Investigative Dermatology*.

[23] Elrayess M. A., Webb K. E., Flavell D. M. (2003). A novel functional polymorphism in the PECAM-1 gene (53g>A) is associated with progression of atherosclerosis in the LOCAT and REGRESS studies. *Atherosclerosis*.

[24] Elrayess M. A., Webb K. E., Bellingan G. J. (2004). R643G polymorphism in PECAM-1 influences transendothelial migration of monocytes and is associated with progression of CHD and CHD events. *Atherosclerosis*.

[25] Popovic D., Nikolajevic Starcevic J., Santl Letonja M. (2016). PECAM-1 gene polymorphism (rs668) and subclinical markers of carotid atherosclerosis in patients with type 2 diabetes mellitus. *Balkan J med Genet*.

[26] Shalia K. K., Mashru M. R., Soneji S. L. (2010). Leucine125Valine (Leu125Val) gene polymorphism of platelet endothelial cell adhesion molecule-1 (PECAM-1) and myocardial infarction in Indian population. *Indian Journal of Clinical Biochemistry*.

[27] Kikuchi M., Looareesuwan S., Ubalee R. (2001). Association of adhesion molecule PECAM-1/CD31 polymorphism with susceptibility to cerebral malaria in Thais. *Parasitology International*.

[28] Robbins F. M., Hartzman R. J. (2007). CD31/PECAM-1 genotyping and haplotype analyses show population diversity. *Tissue Antigens*.

[29] Wei H., Fang L., Chowdhury S. H. (2004). Platelet-endothelial cell adhesion molecule-1 gene polymorphism and its soluble level are associated with severe coronary artery stenosis in Chinese Singaporean. *Clinical Biochemistry*.

[30] Fang L., Wei H., Chowdhury S. H. (2005). Association of Leu125Val polymorphism of platelet endothelial cell adhesion molecule-1 (PECAM-1) gene & soluble level of PECAM-1 with coronary artery disease in Asian Indians. *The Indian Journal of Medical Research*.

[31] Feng Y. M., Chen X. H., Zhang X. (2016). Roles of PECAM-1 in cell function and disease progression. *European Review for Medical and Pharmacological Sciences*.

[32] Wang Y., Sheibani N. (2002). Expression pattern of alternatively spliced PECAM-1 isoforms in hematopoietic cells and platelets. *Journal of Cellular Biochemistry*.

